# Childhood Obesity in Serbia on the Rise

**DOI:** 10.3390/children8050409

**Published:** 2021-05-18

**Authors:** Lidija Marković, Višnja Đorđić, Nebojša Trajković, Predrag Božić, Szabolcs Halaši, Dragan Cvejić, Sergej M. Ostojić

**Affiliations:** 1 Faculty of Sport and Physical Education, University of Novi Sad, 21101 Novi Sad, Serbia; markoviclidija169@gmail.com (L.M.); djordjicvisnja@gmail.com (V.Đ.); sergej.ostojic@chess.edu.rs (S.M.O.); 2 Faculty of Sport and Physical Education, University of Niš, 18000 Niš, Serbia; 3 Serbian Institute of Sport and Sports Medicine, 11030 Beograd, Serbia; predrag.bozic@rzsport.gov.rs; 4 Faculty of Sport and Physical Education, University of Montenegro, 81400 Nikšić, Montenegro; 5 Teacher Training Faculty in the Hungarian Language in Subotica, University of Novi Sad, 24000 Subotica, Serbia; halasi.sabolc@gmail.com; 6 Faculty of Education in Sombor, University of Novi Sad, 25101 Sombor, Serbia; dcvejic@yahoo.com

**Keywords:** childhood obesity, overweight, Serbia, WHO definitions, IOTF definitions

## Abstract

The aim of the study was to examine changes in obesity prevalence among primary school children in Serbia between 2015 and 2019 rounds of the national WHO European Childhood Obesity Surveillance Initiative (COSI-Serbia). Cross-sectional studies were conducted in 2015 and 2019. The nationally representative samples of primary school children were measured for body height and weight, following the COSI protocol. Body Mass Index was calculated, and the IOTF and WHO definitions were used to classify children as overweight or obese. Participants were children of both sexes aged 7.00–8.99 years (*n* = 6105). Significant differences in overweight (obesity included) prevalence between two COSI rounds were identified regardless of definitions applied. According to the WHO definitions, prevalence of overweight and obesity combined increased in 7–9-year-old children in Serbia from 30.7% in 2015 to 34.8% in 2019 (z = −3.309, *p* < 0.05), and according to the IOTF standards, the increase from 22.8% to 30% was registered (z = −6.08, *p* = 0.00). The childhood overweight/obesity rate is increasing in Serbia, which places monitoring and surveillance of children’s nutritional status high on the public health agenda.

## 1. Introduction

Obesity is an alarming global health problem, with more than 107 million children and 603 million adults affected [1]. It is defined as abnormal or excessive fat accumulation that presents a risk to health [2]. Many studies suggest that the prevalence of obesity depends on different factors such as gender, ethnicity, socioeconomic status, and parent’s level of education [3,4,5]. In previous decades, the prevalence of obesity has increased both in children and adults, yet in many countries, the increase is higher in children. As for the European countries, the number of children with obesity seems to have stabilized in the last decades, with substantial regional differences [6]. The highest prevalence rates of overweight and obesity were reported in the Mediterranean region, while in the Atlantic region, the rates were the lowest.

According to available World Health Organization data, overweight (including obesity) prevalence in primary-school children in 35 European countries varies from 9% to 42% among boys and from 5% to 43% among girls, and the obesity prevalence reaches 2% to 21% among boys and 1% to 19% among girls [7]. In Serbia, the overweight and obesity combined rate was reported to be as high as 23% in 6–9-year-old children, with an obesity rate of 6.9%. Therewith, overweight (including obesity) prevalence in Serbian 6–9-year-old children varies from 22.1% to 24.6% among boys, and from 23.1% to 24.3% among girls [8].

Associations between individual health-risk behaviors related to the frequency of consuming food and physical activity and childhood obesity in European countries appeared to be inconsistent, with some health-risk behaviors showing positive and other negative associations with obesity or overweight [9]. However, on the other hand, the authors stated that a combination of health-risk behaviors was consistent in association with obesity or overweight, especially in multiple physical activity-related risk behaviors. Oppositely, physical activity in children, spending time outdoors and engaging in activities such as play improve children’s health and wellbeing [10].

Considering the scale of the problem and numerous adverse health effects of overweight and obesity [11,12,13,14,15,16], it is of vital importance to develop policies concerning prevention, monitoring, and treatment of childhood obesity. In order to provide a European-wide harmonized surveillance system for childhood obesity, the World Health Organization (WHO) Regional Office for Europe launched the WHO European Childhood Obesity Surveillance Initiative (COSI) in 2006. The COSI targets primary school children (6–9 years) since quality data on the nutrition status of this population group are lacking. The initial main outcomes of interest of COSI implementation were anthropometric outcome measures, such as BMI. Family record form implies data on the children’s characteristics and lifestyle behavior, household characteristics, and parental socioeconomic status (educational and occupational level). It was optional and provided by children’s parents or caregivers. School administrators filled in a school record form considering data on a school, classes, environment, and organization in school, also physical activity, nutrition, and promotion of active lifestyle behavior.

Serbia joined the COSI for Implementation Round 4 (2015/2016), intending to develop a surveillance system that would provide reliable, objective, and internationally comparable data on overweight and obesity prevalence among primary-school children. In Round 5 (2018/19) the Serbian national team collected data for the second time in order to identify obesity prevalence changes over four years. Further, obesity trends could be used to inform the public, professionals, and policymakers and helped them to develop new policies for obesity prevention in children.

## 2. Materials and Methods

The research was instigated following the ethical principles, and all procedures were in accordance with ethical standards of the responsible committee on human experimentation (institutional and national) and with the Helsinki Declaration of 1975, as revised in 2008. The present study was performed under the COSI protocol. The study was conducted in accordance with the Declaration of Helsinki, and all procedures were approved by World Health Organization (No. 2018/873491-0). Parents, teachers, and school administrators were fully informed about all study procedures, and active informed written consent was requested from the parents. Verbal consent from the child to participate in the study was obtained on the measurement day.

The first and the second COSI data collection rounds in Serbia were conducted in 2015 and 2019, respectively. Following the COSI protocol [17], cross-sectional design was employed in both rounds, targeting primary school children aged 6.00 to 8.99 years in 2015, and 6.00 to 9.99 in 2019. In both rounds, nationally representative samples were selected through cluster sampling, with the primary school as the primary sampling unit. Schools were selected randomly from the list of public primary schools provided by the Serbian Ministry of Education. Since less than 1% of the target children were enrolled in private or special schools, these schools were excluded from the sampling frame. Stratification by the region, district, and level of urbanization was applied. The minimal planned sample size consisted of 2400 children per each round, as recommended by the WHO European Office.

Trained field examiners performed standardized anthropometric measurements in participating schools. Children were asked to take off their shoes and to remove any heavy objects like wallets and mobile phones before measurement. They were mostly measured in everyday light clothes, with bodyweight additionally corrected for an average weight of the clothes worn (gym clothes −0.15 kg, light clothing −0.35 kg, heavy clothing −0.5 kg). Bodyweight was measured to the nearest 0.1 kg using portable digital scales (Omron BF214, Kyoto, Japan), while height was measured to the nearest 0.1 cm by a portable stadiometer (Seca 213, Hamburg, Germany). 

Body Mass Index (BMI) was calculated as weight (kg) divided by height squared (m^2^). In order to identify overweight and obese status in children, the revised international IOTF cut-offs [18] and the WHO cut-offs [19] for school-age children and adolescents were applied. The WHO AnthroPlus software (Nutritional Survey module) for 2007 WHO growth reference was used to calculate the Z-scores for BMI-for-age [20]. The z score test for two population proportions was applied to test differences in overweight/obesity prevalence in COSI national round 1 and 2. The data were analyzed using the statistical package (SPSS Statistics 21.0, IBM Corporation, Chicago, IL, USA).

## 3. Results

In the first data collection round in 2015, we recruited 5102 children aged 6.00–8.99 years from 42 public primary schools. After a quality check evaluation, 214 children were excluded due to missing or inaccurate data. The final sample consisted of 4861 participants (2386 girls) who entered the final analysis. 

In the second national round in 2019, 3920 children aged 6.00–9.99 years from 57 public elementary schools were approached. Only two schools refused to participate, and 55 schools from all statistical regions in Serbia, and 26 out of 29 districts participated in the study. However, some children were absent on the measurement day, and in some cases, parental consents were not provided, therefore, 563 children were excluded. The next 178 children were ruled out because of missing or inaccurate data, so the final sample consisted of 3179 participants (1506 girls). The basic characteristics of the 2015 and 2019 samples are presented in Table 1. In order to compare childhood overweight and obesity prevalence in 2015 and 2019 rounds, only data of overlapping age groups: 7.00–7.99 and 8.00–8.99 years were used. Prevalence rates for overweight and obesity in 2015 and 2019, using the IOTF and WHO definitions, are presented in Table 2.

Overall obesity and overweight ratio have increased between two rounds in 8–9-year-old girls regardless of the definition applied, and boys of both ages when the IOTF criteria were used. At the same time, similar trends were observed in 7–8-year-old girls, and boys of both ages according to the WHO criteria (Table 2). The rates of overweight and obesity are generally higher when the WHO definitions are applied in comparison to the IOTF. This particularly refers to the estimates of obesity, with the WHO numbers being up to nine percentage points higher. 

According to the WHO and IOTF cut-offs, the lowest obesity rate was observed in 8–9-year-old girls in 2015 (7.9% and 6.2%, respectively). However, the highest obesity estimates differ depending on the criteria applied. According to the WHO criteria, the highest obesity rate was observed in 8–9-year-old boys in 2019 (17.1%), while the IOTF standards pointed out 8–9-year-old girls measured in the same round (10.9%).

As for the sex differences, the tendency of higher obesity prevalence in boys was identified in both rounds, when the WHO definitions are applied. For example, in the 2015 round, the prevalence of obesity was almost two times higher in 8–9-year-old boys in comparison to the girls of the same age (15.7% and 7.9%, respectively). In the 2019 round, the sex differences were less pronounced. On the other hand, when IOTF definitions are used to estimate childhood obesity, the pattern no longer exists. As for the overweight prevalence, the numbers are higher in girls in almost all cases, regardless of the criteria employed.

Finally, z-test for proportions revealed significant differences in overweight (obesity included) prevalence between two COSI rounds. According to the WHO cut-off points, the prevalence of overweight and obesity combined increased in 7–9-year-old children in Serbia from 30.7% in 2015 to 34.8% in 2019 (z = −3.309, *p* < 0.05). Provided that the IOTF standards are used, the increase in overweight and obesity rate is even more pronounced, from 22.8% in the first round to 30% in the second round (z = −6.08, *p* = 0.00).

## 4. Discussion

Serbia joined COSI in 2015, at the fourth data collection round, and also participated in the fifth round in 2019. The increase in both overweight and obesity in Serbian children aged 7–9 years has been identified, while in most of the COSI participating countries, a decrease or stable prevalence were reported within a ten-year time frame [7]. A recent meta-analysis suggests that although the prevalence of childhood overweight and obesity is very high in European countries, in most cases, trends have stabilized from 1999 to 2016 [6]. However, the increase in the prevalence of overweight and obesity was recorded in some Mediterranean countries [6]. Contrary to our findings, the same study pointed out that the prevalence of overweight and obesity is higher in girls in most European countries. In accordance with our results are the COSI data collected in the 2015/2017 round, where the prevalence of obesity was higher in boys in nearly all countries, and the prevalence of overweight was higher in girls in two-thirds of countries [7]. This was confirmed in the neighboring country, using COSI methodology, showing that Croatia has one of the highest prevalence of childhood overweight and obesity among European countries, which was more frequent among boys [21]. One more neighboring country (Hungary) showing the COSI results from 2016 is facing overweight and obesity as emerging problems [22]. 

The unfavorable trend in childhood obesity might be related to the lack of health-promoting lifestyle habits in Serbian children and youth, where only 8% of early adolescents in Serbia choose a diet low in fats, 17% limit use of sugars and sweets, and 20% eat recommended servings of vegetables each day [23]. Only 45% of Serbian adolescents follow a planned exercise program, 43% exercise vigorously for 20 or more minutes at least three times a week, 40% take part in light to moderate physical activity, and 29% take part in recreational physical activity [23].

The high prevalence of childhood overweight and obesity in Serbia confirmed in the current study, along with the previous findings that overweight (obesity included) rates are lowest among children from well-developed communities [8], suggest that Serbia is in the third stage of obesity transition, like the USA and many European countries [24]. However, it should be noted that there were small differences in the reference system, especially for estimating obesity, with the WHO numbers being up to nine percentage points higher. The difference between IOTF and WHO reference system was documented recently in 5–17 years old children [25]. The authors explained these differences with population variations in the pattern of BMI with gender and age between nations and also with the time of data collection.

According to Jaacks et al. [24], reducing the prevalence of adulthood obesity might be related to reducing obesity in children in the first place, therefore, regular monitoring and assessment of children’s nutritional status and nutritional environments should be considered as a public health priority. This was confirmed in Spain that reached a stabilization situation regarding obesity by the implementation of public measures to prevent childhood obesity [26]. Moreover, some countries with the highest prevalence that were involved in COSI project, showed a decrease of the BMI z-score with public health measures taken after the first round [27]. However, stabilization in nutritional status could be transitory and could appear easy again in the future. Therefore, schools should integrate educational procedures relying on critical thinking, nutrition, and physical activity via diet and school curriculum and provide suitable areas for recreation, sport, and outdoor play, parental and community participation, and restrain commercialization of processed food [28]. Moreover, besides schools, coordinated efforts from multiple governmental sectors and institutions are essential in order to decrease, stabilize, or prevent obesity.

## 5. Conclusions

The childhood overweight/obesity rate is increasing in Serbia, which should place monitoring and surveillance of children’s nutritional status high on the public health agenda. Comparing the incidence of obesity in the world and in our country, we see that our country follows the trend of increasing obesity. Based on the presented results of the research, we see that it every third child in our country is overweight or obese, which is very worrying because, according to research, this trend of obesity in children will grow even more. Serbia, as a developing country, follows the trend of obesity in all structures of society. Therefore, the entire state and numerous organizations should be involved, and a lot more should be invested in order to stop this trend of obesity worldwide. 

## Figures and Tables

**Table 1:** Basic characteristics of the final samples in Serbian COSI 2015 and 2019 rounds.

	2015	2019
Final sample size	4861	3179
Girls	49.1% (2386)	47.4% (1506)
Boys	50.9% (2475)	52.6% (1673)
6-year-olds	16.9% (824)	0.7% (22)
7-year-olds	48.2% (2341)	31.1% (989)
8-year-olds	34.9% (1696)	33.9% (1079)
9-year-olds	-	34.2% (1089)
Age (years)	7.7 ± 0.6	8.5 ± 0.8
Weight (kg)	28.4 ± 6.3	31.7 ± 7.9
Height (cm)	129.7 ± 6.8	134.1 ± 7.8
Body mass index (kg/m^2^)	16.7 ± 2.6	17.4 ± 3.0

**Table 2:** Prevalence of overweight and obesity among Serbian children aged 7–9 years in national COSI round 1 (2015) and 2 (2019) using the WHO and IOTF definitions.

	WHO (2015)				WHO (2019)				IOTF (2015)				IOTF (2019)			
	Overweight		Obese		Overweight		Obese		Overweight		Obese		Overweight		Obese	
	%	95% CI	%	95% CI	%	95% CI	%	95% CI	%	95% CI	%	95% CI	%	95% CI	%	95% CI
Girls																
7–8 years	19.1	16.9–21.5	9.7	8.0–11.5	18.8	15.3–22.7	10.7	8.0–14.0	17.0	14.9–19.2	7.3	5.9–8.9	19.2	15.7–23.2	9.4	6.9–12.5
8–9 years	19.5	16.9–22.5	7.9	6.1–10.0	23.8	20.2–27.6	13.2	10.4–16.4	16.5	14.0–19.3	5.3	3.8–7.0	23.2	19.6–27.0	10.9	8.4–13.9
Boys																
7–8 years	18.7	16.5–21.0	13.9	12.0–16.0	20.5	17.2–24.1	15.1	12.2–18.4	15.9	13.9–18.1	6.2	4.9–7.8	18.3	15.1–21.8	9.4	7.1–12.2
8–9 years	17.8	15.3–20.4	15.6	13.3–18.2	19.2	16.0–22.7	17.1	14.0–20.4	15.9	13.5–18.4	6.9	5.4–8.8	19.7	16.5–23.3	9.9	7.5–12.7
All	18.8	17.6–20.0	11.9	10.9–12.9	20.6	18.9–22.4	14.2	12.7–15.8	16.3	15.2–17.5	6.5	5.8–7.4	20.1	18.4–21.9	9.9	8.7–11.3

WHO, WHO obesity/overweight definition; IOTF, IOTF obesity/overweight definition; 95% CI, 95% confidence interval; y, years.

## Data Availability

The data presented in this study is available on request from the corresponding author.
